# “A disease that god has given me” patients and caregivers’ perspectives on diabetes in southeastern Tanzania

**DOI:** 10.1186/s12889-023-15147-3

**Published:** 2023-01-31

**Authors:** Emmy Metta

**Affiliations:** Department of Behavioural Sciences, School of Public Health and Social Sciences, Muhimbili University of Health and Allied Sciences, P.O. Box 65015, Dar es Salaam, Tanzania

**Keywords:** Diabetes causes, Diabetes susceptibility, Diabetes severity, Diabetes prevention

## Abstract

**Background:**

Prompt diagnosis and appropriate management of diabetes has the potential of improving survival and patient health outcomes. Yet many diabetes patients present themselves to health facilities at an advanced stage of the disease which complicates its management. Individual perceptions about diseases are known to play a critical role in informing responses and actions including seeking health care and self-care practices. However, little is documented in Tanzania regarding the perspectives of diabetes patients and their caregivers about the disease especially in rural settings.

**Methods:**

We conducted 26 in-depth interviews involving 19 diabetes patients and 7 diabetes patient caregivers to explore in detail their perspectives on diabetes as a disease. Data was analyzed using thematic analysis with the help of NVivo9.

**Results:**

Both patients and caregivers expressed mixed perceptions on diabetes causes. In addition to heredity, and the failure of the pancreas to function well, lifestyle factors including lack of physical activity and eating too many sugary and oily foods were common reported causes. However, none of the participants were clear on the mechanisms between the perceived causes and the actual occurrence of the disease. Perception on susceptibility to diabetes was low even among participants with the disease as they reported not having ever thought of getting the condition before they were diagnosed. Some caregivers expressed worry and fear on their susceptibility to inheriting diabetes from their relatives who had the condition. Diabetes was perceived as a severe and life-threatening condition that can easily cause death if not well managed. Participants indicated uncertainty on its prevention.

**Conclusion:**

This study shows mixed perspectives on the causes, susceptibility, severity and prevention of diabetes which were informed by the participants’ limited knowledge and awareness about the disease. Interventions to strengthen responses to diabetes, which include buy-in from the patients and their caregiver’s perspectives are essential to improve prevention, early diagnosis and appropriate management in rural settings.

## Introduction

Diabetes is one of the main global killer diseases that place the worlds’ public health into a tragedy. In 2021, roughly 537 million people had diabetes globally with an estimated rise to 643 million by 2030, and to 783 million by 2045, the majority of these being people with type 2 diabetes. Diabetes accounts for 1.6 million of global premature deaths annually [1]. In Sub Saharan African (SSA) countries, diabetes is rising at an alarming pace and it is estimated that it will affect 55 million people by the year 2045, up from 15.9 million estimates made in 2017 [2]. This region is home to the highest mortality from diabetes and the highest rate of an undiagnosed diabetes population compared to the rest of the world [3]. During 2021, about 416,000 deaths that occurred in the SSA region were associated with diabetes [3]. The upstage of diabetes is happening alongside the ongoing challenges of predominating infectious diseases and other health conditions related to poverty [4, 5]. As a consequence of prevalent sedentary lifestyles, rapid urbanization, and modified diets, the rising burden of non-communicable diseases (NCDs) including diabetes is anticipated to surpass that of communicable diseases by the year 2030 [4].

In Tanzania, the impact of diabetes among other NCDs is devastating. According to the International Diabetes Federation (IDF) 2021 report [6], a total of 2, 288,000 Tanzanian adults are living with diabetes. According to the said report, diabetes prevalence is 10.3% which is higher than the 9.1% prevalence reported in 2012 [7] and is a massive increase from the 2.5% prevalence reported in 1984 [8] among people of the same population group. Despite this shocking statistics, the reported prevalence could be underestimated given the established fact that more than 80% of Tanzanians are living with undiagnosed and untreated diabetes and are unaware [9, 10] due to limited community knowledge about the condition [11]. Evidence on the prevalence of diabetes in rural settings is limited, however, rural areas are reported to have higher prevalence of people with glucose impairment, a risk factor for developing diabetes [12, 13]. Literature tells us that the prevalence of diabetes like many other NCDs increases with population growth, aging, urbanization, sedentary lifestyles and changing dietary patterns [14], all of which are characteristic in Tanzanian settings.

Diabetes is an overwhelming long-lasting disease silently tormenting the social, psychological and wellbeing of patients and their families. The condition requires prompt and appropriate management to prevent the development of complications and to improve patient health outcomes and wellbeing. Suitable diabetes self-care and management is associated with better blood glucose levels and improved quality of life among diabetes patients [15]. However, limited awareness on the epidemiology of diabetes including its prevalence, modifiable risk factors such as diet and obesity coupled with the high rate of undiagnosed diabetes, challenges efforts to address diabetes in Africa [3] and Tanzania in particular [12, 13]. In Tanzania, many people with diabetes present to health facilities for diabetes care when the condition is already at an advanced stage [16, 17]. This underlines the need for locally generated information on the perspectives related to this surging condition to provide insights that will inform better ways to design and engage community members on its prevention and encourage prompt management and control.

Several studies report the importance of early diagnosis of diabetes and prompt initiation to treatment [18–20] so that people with diabetes can live longer and healthier lives if the condition is promptly diagnosed and appropriately managed. Studies also report several factors deterring diabetes patients from accessing diabetes care timely and continuing with care [17, 21, 22]. Descriptions on how diabetes patients view their treatment and self-care practices, and experiences on living with diabetes are widespread [22–25]. However, studies to understand patient perceptions on the causes of their diabetes condition, their perception of risk, severity and prevention against the condition are limited. The few studies done focused on understanding how diabetes is viewed in the general community and the risk factors. A study in Indonesia, for example, reported that diabetes patients are blamed for getting the condition with others believing that they cannot get diabetes [26]. This indicates a low-risk perception which is likely to hinder success on efforts to promote prevention of the disease and its prompt management. In Kenya, poor knowledge on the causes and effects of diabetes was established in more than 70% of the study participants [27]. Negative perceptions towards diabetes are prevalent in many different settings, especially among rural communities. Yet, studies to understand how communities with diabetes perceive the diabetes are limited especially in Tanzanian rural settings. It is important to generate more information on people’s perceptions of the disease as this will inform targeted interventions aimed at promoting better health seeking behavior and early diagnosis, and improving preventive and self-care practices and diabetes-related health outcomes.

Individual perceptions about the disease play an important role in informing responses, including treatment seeking and continuity with care [21, 26]. According to the Health Belief Model (HBM), individuals are likely to change their behavior if they perceive something as a personal threat that can lead to an illness or disease (perceived risk to diabetes) and that the said illness or disease is likely to yield negative consequences (perceived severity). The HBM model postulates that the higher the perceived severity the more likely individuals will adapt the recommended behaviors to circumvent the negative consequences. The model envisions individual decisions to participate in diabetes preventive measures as being informed by several factors including their perception on the disease and its impact, as well as on the benefits of undertaking measures [28]. As such, understanding individual perceptions and beliefs is necessary for informed strategies to prevent, control [29] and increase awareness of the health risks of a disease. Peoples’ perception of a disease is formed by their views and ideas about its cause, susceptibility, preventive measures, and the likely threat from the disease [30]. Previous studies provided necessary information on the prevalence of diabetes, self-care practices and adherence to medicines [12, 31] however, they do not help us to understand more about the patients and their caregivers’ perspectives on diabetes. A dyadic approach is required to understand patient and their caregiver perspectives on a disease because this is critical in shaping self-care practices especially for chronic conditions [32, 33] including diabetes that requires long-term medical attention and care. The caregiver of a person suffering from NCDs undertakes multiple caring roles ranging from helping patients to access basic health care needs and making treatment decisions, to providing emotional and psychological support that have a bearing on a patient’s quality of life and wellbeing in general. This study therefore aimed at gaining a deeper understanding of the perceptions of diabetes patients and their caretakers on diabetes causes, perceived susceptibility, perceived severity and preventive measures. Such information is fundamental for designing effective prevention and self-care interventions that are critical for controlling NCDs such as diabetes and improving diabetes health-related outcomes.

## Methods

### Design

The study used a qualitative research design and employed in-depth interviews with diabetes patients and their caregivers that explored their perspectives on diabetes. Qualitative research was the most suitable approach as the study intended to elicit participants’ experiences and views [34, 35] about diabetes. The use of in-depth interviews (IDI), which are known to elicit a detailed understanding of the phenomena in question [36], provided an opportunity for a deeper understanding of the participants’ views and experiences which was necessary in addressing the study objective.

### Recruitment of study participants

Study participants were adults with diabetes and their caregivers who were purposefully recruited from the diabetes clinic at the district and from a semi-urban village surrounding the clinic. The diabetes clinic provides services to people from different parts of the district. Permission was obtained from the hospital management to access people with diabetes through the diabetes clinic. The clinics’ wider service provision coverage provided an opportunity for the study to also access and recruit people who were visiting the clinic from rural and remote villages. The research team in consultation with the clinic Nurse purposefully identified participants from remote villages who had been diagnosed with diabetes for a period of not less than six months to the time of the study. The six-month period was considered as being long enough for the participants to have conceptualized the disease and gained fairly sufficient experience on it. The identified participants were contacted after they were done with their clinic consultations and were informed about the study and its objectives in detail. They were also given opportunity to ask questions or seek clarification from the research team before asked about their willingness to participate in the study. The patients were also asked to identify individuals who were taking care of them. Some of these caretakers were purposefully recruited for the in-depth interviews. However, only those who expressed willingness and provided consent were invited to participate in the in-depth interviews. The interviews for both diabetes patients and their caregivers took place at a private place close to the diabetes clinic that was specifically identified for that purpose to ensure privacy and confidentiality. They were conducted separately to allow the individual participants more freedom to express their perspectives on the disease.

In the semi-urban village, the research team in consultation with leaders of the area purposefully identified people known to have diabetes and who have had their clinic cards reviewed to confirm diabetes diagnosis before recruiting them in the study. The identified individuals were given detailed information about the study and asked whether they were willing to participate. Different entry points were used to avoid recruiting people of the same social networks. The in-depth interviews with participants from the semi-urban village were conducted at the participants’ households. The recruitment of participants at both sites was stopped after data saturation was reached.

### Data collection and analysis

Data collection activities were conducted by a team of two Social Scientists; the author and a Research Assistant. An in-depth interview guide which was refined after the pilot study was used to guide the interviews. The author conducted all 26 interviews in Kiswahili, a language spoken by all study participants. A total of 19 diabetes patients (10 females and 9 males) and 7 caregivers (4 females and 3 males) were interviewed. The interviews were 45–60 minutes long and were audio recorded. The Research Assistant transcribed the audio recorded files verbatim within 48 hours of the time the interviews were conducted and saved the transcripts as word files.

The author then reviewed the transcripts against the audio recordings to ensure their quality and accuracy before they were imported into qualitative data management software (QSR Nvivo 9) for analysis. The author read and re-read each transcript to familiarize with the data and identify initial codes. This was followed by the development of inductive and deductive codes and the writing of the thick description. The codes were categorized into themes and family codes following the principles of grounded theory. Even though the data on the perspectives of diabetes provided by people with diabetes and their caregivers were fairly consistent, the results of this study are not presented separately by type of participant, instead they are used to describe different themes that emerged from the data.

## Results

### Characteristics of the participants

The diabetes patients’ age ranged from 35 to 75 years while that of the caregivers ranged from 21 to 62 years. The majority of participants were married and had between 1 and 8 children. The education level of the participants ranged from no education to some secondary level education, with the majority being standard seven leavers with farming as their main economic activity, see Table 1. Most have had diabetes for more than 5 years and were on insulin treatment.

**Table 1:** Sociodemographic characteristics of the study participants

UNIQUE IDNO	Sex	Age	Marital status	No. of children	Education Level	Years with diabetes	Economic activity
Patient 010	F	61	Window	8	From2	6	Farmer
Patient 020	F	39	Married	1	St. 7	5	Farmer
Patient 030	F	58	Married	2	St. 7	3	Farmer
Patient 040	F	52	Married	5	Form4	5	Teacher
Patient 050	M	70	Married	7	St.8	4	Farmer
Patient 060	F	41	Married	3	St.7	6	Farmer
Patient 070	F	54	Widow	4	St. 7	4	Farmer
Patient 080	M	57	Married	6	St. 7	6	watchman
Patient 090	M	58	Married	6	St. 7	10	Farmer
Patient 010	M	75	Married	7	ST. 3	13	Nothing
Patient 011	M	73	Widower	4	St. 4	7	Nothing
Patient 012	F	65	Single	0	St. 4	11	Nothing
Patient 013	F	40	Widow	2	St. 4	8	Farmer
Patient 014	F	75	Widow	4	St.0	12	Nothing
Patient 015	F	36	Married	6	St.7	9	Farmer
Patient 016	M	63	Married	6	St 0	23	Watchman
Patient 017	M	42	Married	3	Form2	7	Tailor
Patient 018	M	56	Married	8	Form4	8	Farmer
Patient 019	M	35	Married	3	St.0	5	Watchman
Caregiver 020	F	44	Married	1	St. 7		Petty trader
Caregiver 021	M	62	Married	2	St. 4		Farmer
Caregiver 022	M	21	Single	0	St. 7		Farmer
Caregiver 023	F	59	Married	7	St. 4		Petty trader
Caregiver 024	M	45	Married	6	St. 7		Farmer
Caregiver 025	F	60	Married	6	St. 0		Farmer
Caregiver 026	F	47	Married	6	St. 7		Farmer

### Perceived diabetes causes

All study participants were not forthcoming in explaining the causes of diabetes with “I don’t know” being a common response. It is only after probing that patients expressed different views on what they perceived as the cause of the diabetes condition they are suffering from. The majority of participants with diabetes insisted that they had no idea what caused the disease claiming that they just found themselves with diabetes and that they believed it is a disease from God. When explaining the cause of diabetes one of the patients reported the following:

*“I mean, I have no idea where it (diabetes) came from... I just found myself with it.… I believe it is a disease that God has given me… I just have to accept it, what else can I do”* 61 years, female participant with diabetes.

Some participants with diabetes expounded on the causes of diabetes by referring to what they are being told at the health facility during their visits, that diabetes is caused by a *“failure of the pancreas to do its work properly”* however, most of them do not understand how the pancreas fails and what causes it to fail.

“…. *every time I tried to ask the doctor on what is the exact cause of this sugar disease disturbing me he says …this occurs due to a failure of the pancreas to do its work properly, I don’t know…. it does not make [give] enough insulin (mmh) …. that is what the doctor told me but mmh I don’t real understand how it fails and what causes that failure?”* 36 years, female participant with diabetes

The idea that diabetes can be passed on from one generation to another was raised by both patients and caregivers. Participants recalled questions that were asked at the health facility the first time they were diagnosed with diabetes. It was clarified that it is common when doctors find one has diabetes to ask questions like “*in your family or clan is there anyone with diabetes?”* This questioning to establish the existence of the condition in the family and or clanship made some participants believe that diabetes could be inherited from the clanship. as understood by this participant:

*“I think it is inherited, in my family, my father had diabetes and my grandmother had it too (mmh) I understood when the doctor asked me … because it is inherited through one generation and blood”* 42 years, male participant with diabetes

Several other causes of diabetes were mentioned by both patients and caregivers as probing continued. They included lack of physical exercise, eating foods with too much oil and too much sugar, or sweet things. However, some participants with diabetes voiced their concern on eating food with too much oil or sugar as causes of diabetes because even children suffer from the disease. As one of the participants with diabetes said:

*“Aah many people think that diabetes is caused by eating oily foods and sugary things but I am asking myself what about children? Some of these children are as young as four years and you find they have diabetes (mmh)….for adults like me one may say I have eaten a lot of sugary and oily foods for a long time ….but what about those children ….how come they also have this ‘sugar’ disease?”* 58 years, female participant with diabetes

Sugar as a cause of diabetes was widely discussed in the study with concerns from both participants with diabetes and caregivers. Some of the participants with diabetes claimed that they were not used to like or eat sugary foods and rarely took tea with a lot of sugar but were diagnosed with diabetes. Others were concerned that in their families they cook and eat from the same pot as a family but it is only them who were diagnosed with diabetes. This raised doubt on the possibility of there being other causes of diabetes. As this participant explained:

“*I hear it is caused by eating too much sugar, the sweet things that many people like….but one thing I wonder is that in my family we normally cook and all eat from the same pot …I used to eat what the rest of the family ate but it is only me who was diagnosed with diabetes …… that is why I wonder whether it is really about sugary foods only or whether there is something else causing this disease (mmh) because how come it is only me who was diagnosed with the disease”* 70 years, male participant with diabetes

To some of the caregivers the sugar-diabetes cause relationship was even more challenging. These caregivers said they wondered a lot as to how sugar can cause an illness to a person. They continued arguing about how sugar is the cause of diabetes and at the same time it is a treatment of the same. This was seen to cause them confusion especially because when people with diabetes are low on energy, they are advised to drink a solution with a lot of sugar. Three of the caregivers reported to have made a sugar solution many times and given it to their relatives with diabetes when they saw them looking weak or when they became unconscious. One of the caregivers when expressing the dilemma he is facing explained the following:

*“I heard someone saying it (diabetes) is caused by eating too much sugar… now I wonder what happens then? Because if it is said to be caused by eating too much sugar how come after a person is diagnosed with the ‘sugar disease’ and… if you see them loosing energy you are told to given them a water solution with plenty of sugar? … for instance these days my father is not allowed to take tea with sugar but when he faints we are told to prepare a solution with plenty of sugar and give him so that he can regain consciousness … they have also restricted him from eating salty foods….. does salt also cause “sugar disease”? I don’t really understand how this disease comes about, I think it is a disease that God decided to happen to him!”* 21 years, male caregiver

### Perceived susceptibility to diabetes

None of the study participants mentioned ever having thought they would get diabetes in their life span even among participants with diabetes. All participants with diabetes said the first time they knew about diabetes is the day they were diagnosed with it. They also said it is likely that many people in the community have the condition but they do not know. The high number of people with diabetes at the diabetes clinic was seen as an indication that the disease is widespread. One of the participants with diabetes noted that recently he has been seeing new faces at the diabetes clinic including children, which is different from how it used to be as he explains below:

*“I see nowadays many people are getting it (diabetes) (mmh) and if you want to confirm this just look at the diabetes clinic there ….the number of people these days is huge …. most of them seem to be newly diagnosed because I have never seen them before, and some of those I used to see when I started the clinic I don’t see them anymore (mmh) What I see is many new faces including children… some are quite young and are brought to the clinic with this same illness, this was not common but nowadays everyone gets it”* 65 years, male participant with diabetes

Additionally, the presence of posters and stickers on diabetes at the diabetes clinic was cited as an indication on the increased prevalence of the disease in the population hence the need to raise awareness about it within the community.

*“..during those days the hospital did not provide many diabetes services because there were a few patients (mmh) at the most you would find them providing hypertension (BP) services (mmh) that is what they were mostly dealing with …but these days there are many people with diabetes... yes many people are getting it and the number keeps rising that is why they made special clinic days for people with diabetes only… that alone is enough for one to tell that there is an increase in the number of people with diabetes here (mmh) eeh*” 67 years, male participant with diabetes

Many caregivers were hesitant to state their perceived susceptibility to diabetes. A few said that if it is sugar that causes the condition everyone is susceptible to getting diabetes because everyone takes tea with sugar. Additionally, the idea that a doctor asks patients on whether they had a relative with diabetes or not during their consultation at the health facility was seen to cause worry and fear among some of the caregivers. Talking about their likelihood of getting diabetes one of the caregivers said:

*“I guess I may also get it …because when you go to the hospital for consultation, the doctor asks you if you have a relative with sugar or blood pressure or Asthma?......now as you know my mama has it… so I don’t know who will inherit it and I am the one who is taking care of her but I don’t know how I should protect myself so that I won’t get it”* 41 years, female caregiver

### Perceived severity of diabetes

All study participants instinctively expressed that diabetes is a severe and life-threatening condition and that a person with diabetes can easily die if appropriate action is not taken. Participants were of the opinion that diabetes requires one to properly manage and monitor the sugar level in their body to minimize chances of severe consequences. As one of the participants attested:

*“if not well managed and monitored what else can one expect other than death, diabetes kills very easily if the sugar level is not well managed (mmh) you will simply die before your day…., you know what (mmh) when the sugar level is too low sometimes you lose consciousness and if there is no one around to give you that water with sugar … you can simply die (mmh) yeah they will just find one is no longer there (mmh) …that is why a person with diabetes needs to be very careful with their medication and monitor the sugar level in the body to reduce the possibility of such consequences*” 70 years, Male participant with diabetes

Loss of body parts is another reported consequence of diabetes severity. Study participants voiced their concern that people with diabetes are susceptible to losing some of their body parts including legs and toes due to wounds that do not heal. When a person with diabetes has a wound, it may not heal instead it decomposes to the extent that the part affected needs to be amputated. This is the reason why people with diabetes are advised to protect themselves from getting injuries and to use their medication properly so that they can continue to be healthy:

*“You know sugar is not a disease to play with (mmh) people are losing their body parts as a result of this disease (mmh) I mean you are supposed to protect yourself from getting any kind of injury and use your medications properly if you are to continue being healthy because when your sugar level rises in the body even a little wound like this [showing a finger] can turn into a big one …. I saw this in my friend who we used to meet at the clinic. She had a wound that led to her toes being cut off, the wound was decomposing and giving an awful smell.... that is why I see it is very important to use medication and follow recommendations properly (mmh) one should not stop because that is life (mmh) eeh”* 54 year, female participant with diabetes

Similar sentiments were expressed by caregivers who noted that people with diabetes need to be extra careful and whenever possible to avoid activities that may lead into injuring themselves. To reduce the chance of injuring their toes, health care workers’ advice patients not to walk barefoot. All caregivers reported being reminded to ensure that their relatives with diabetes wore either slippers or sneakers as often as they can especially when engaging in physical activities.

### Perceived diabetes prevention measures

Many study participants said they don’t know how one can protect themselves from getting diabetes. Some of the participants with diabetes were of the opinion that people can protect themselves from getting diabetes by limiting their sugar intake and refraining from eating sweet things. This knowledge is in line with what they are being advised at the health facility during their routine clinic visits, that they should avoid eating foods with a lot of sugar.

*“…limiting the consumption of sugary foods can help to protect one from getting diabetes that is what they tell us.. to stop consuming foods with a lot of sugar (mmh) because that is what increases the sugar level in the body until it reaches a stage that can cause problems like this”* 61 years, female participant with diabetes

On the other hand, several participants with diabetes and caregivers noted that for people to know how to protect themselves from getting diabetes they first need to know what causes the disease and how it gets into the human body. Without knowledge on the cause of diabetes it might be challenging for them to know how to prevent it. One first needs to know what causes the disease before working on how to prevent it:

*“Until one knows exactly what causes diabetes and how it gets into the human body eeh, like how we know malaria is caused by mosquitoes and its prevention is to protect ourselves from mosquito bites…... But for diabetes - - we are very blind .... we know nothing … we first need to get knowledge about this disease (diabetes) what causes it; how it gets into the body; and what to do to protect ourselves from getting it. That will help us a lot even now to protect our families from getting diabetes (mmm) ehee”* 57 years, male participant with diabetes

Furthermore, few participants with diabetes believe that physical exercise and drinking sufficient water are among measures to prevent one from getting diabetes. The logic behind this belief is that when one exercises, they sweat and that sweating helps in expelling dirty substances including the excess sugar from the body. Drinking sufficient water aids in removing toxins from the body and when done together with exercise it helps to protect one from getting many diseases including diabetes.

## Discussion

This study aimed to explore perspectives on diabetes from people with diabetes and caregivers of people with diabetes. The qualitative approach used provided participants the opportunity to reflect on diabetes as a disease and express their feelings on what they believe to have caused it, share their perceptions on their vulnerability to the disease, the severity of it and preventive measures that can be applied to avoid getting diabetes. Studies on perspectives of people with diabetes and caregivers on the conditions of the disease from a Tanzanian setting are limited.

In the study, people had mixed opinions on what causes diabetes. The majority of participants with diabetes were still uncertain about what causes diabetes despite living with the condition for more than 5 years. Views such as “I don’t know”, “I have no idea”, “a disease God has given me”, “God decided for this to happen”, “it is inherited through one’s clan”, were commonly mentioned before participants stated other causal factors for diabetes. Less clarity on the causes of diabetes among patients and their caregivers has also been reported in Kenya [37] and other settings [26, 38]. The tendency for people with diabetes to associate diabetes with God was also observed in Indonesia where it was stated that “diabetes is a gift from God” [39]. The uncertainty on diabetes causes in this study calls for the need for comprehensive health education not only for people with diabetes but also people in the general community so as to bridge the knowledge gap on diabetes causes along with other non-communicable diseases. This is important because perceptions that people attach to the causes of a disease are known to have implications not only on the type of health-related behaviors practiced and the mechanisms employed as responses, but also on disease outcomes [25] and general patient wellbeing.

In the study, participants attributed diabetes causes to heredity or genetic factors passed on through one’s clan and family. Similar observations were reported by Al-Ghamdi [40] where study participants linked diabetes with heredity. The association of diabetes as an inheritable disease may have implications to public initiatives on prevention and early detection of the disease since people with no history of people with diabetes in the family may believe that they are not at risk of getting it. This underlines the need for strengthened educational and preventive interventions for diabetes with more focus on modifiable risk factors, while highlighting that only a small proportion of people with diabetes get it from genetic and family factors.

Eating too many sugary and oily foods was reported in this study as the cause for diabetes although participants showed ambiguity on the link between food and diabetes. Expressions that all family members eat the same food cooked from the same pot but not all were diagnosed with diabetes surfaced in the study. Others mentioned “failure of the pancreas to function well” as the cause of diabetes though they were not clear on the mechanism involved. The observations in the study may reflect the fact that participants with diabetes and their caregivers were asked to talk about what they thought caused diabetes. Study participants would generally have had discussions with their medical practitioners in an attempt to understand the cause of the disease and may have tried to identify a specific cause for their own illness. The association between food consumption and the emerging non communicable diseases is widely reported in the literature [41–43]. For example, high consumption of fats especially trans-fats and saturates are commonly implicated in the cause of insulin resistance, glucose intolerance and poor diabetes health outcomes [44]. Alternatively, a diet rich in whole grains, cereals, high fiber products and non-oil seed pulses is reported as beneficial in halting risks to diabetes as well as in supporting its management [45, 46]. The existing level of awareness on the relationship between food and diabetes in the study presents an opportunity for policy makers and programmers to tap into and tailor interventions to improve dietary awareness among people with diabetes, caregivers and the general community. It is important for people to make informed decisions on dietary patterns and consider it as a critical aspect not only for secondary and tertiary prevention among patients but also for primary prevention among those who do not yet have the disease.

None of the participants with diabetes in the study reported to have had considered themselves susceptible to getting diabetes and only learned about the condition from doctors’ post diagnosis. These results align with those of other studies in Tanzania [11] and across many Sub-Saharan African (SSA) countries [3, 47–49] and other settings [50] where people with diabetes were not knowledgeable of their diabetes condition until they were diagnosed. Medical personnel are generally reported as the main source of information on health issues including disease prevention and their proper management [51]. However, given the emerging nature of some non-communicable diseases including diabetes, these professionals may lack adequate training on certain diseases [52, 53] to be able to counsel patients about the disease, required life style modifications and other aspects of self-care [52]. There is a need to strengthen health system capacity to offer responsive services to suit patient needs including information related to emerging NCDs such as diabetes to improve people’s understanding of the disease and inform better responses. This should as well include raising awareness about diabetes and other NCDs among the general population on its causes, recommended preventive measures and the consequences of the chronic condition, and encourage adaptation of lifestyle changes.

In the study, diabetes was perceived as a severe and life-threatening disease of which a person can easily die of if not appropriately managed. Similar observations were reported elsewhere [25] where diabetes was reported as a life-threatening disease with multitude consequences. The perceived severity of illness has been associated with higher chances for positive self-care practices and adherence to treatment regimens among people with diabetes [25, 54]. However, assessing the likelihood of perceived severity to enhance adherence to diabetes self-care was not within the mandate of the current study. Studies to establish the association between patients’ perceived severity and adherence to self-care practices in rural settings could yield useful insights for informing strategies geared towards improving adherence to self-care practices and patient health outcomes and wellbeing in general.

Literature shows that diabetes is a chronic lifelong disease with severe consequences to the patients and their caregivers [55]. However, it also shows that type 2 diabetes can be prevented and its onset delayed with adherence to healthy eating, regular physical activity, maintaining normal body weight, and avoiding tobacco use [56]. Awareness of diabetes preventive practices was limited in this study where a few participants mentioned limiting the intake of sugary or sweets foods, increased physical activity, and drinking sufficient water, among measures to protect oneself from getting the disease. Dietary habits and a sedentary lifestyle are implicated for the surging incidences of diabetes in developing countries [14]. The views expressed on food and diabetes in the study could serve as a starting point for strengthened communication and improved dissemination of knowledge on diet and lifestyle modifications in relation to diabetes. This will help to provide a clearer understanding of, for example, what it is really meant by a healthy diet in the context of preventing or delaying diabetes among other NCDs. On the other hand, the believe that drinking sufficient water could prevent one from getting diabetes may need further follow-up to establish the mechanisms of change and generate plausible evidence on the role that drinking water plays as a protective factor in diabetes prevention.

Although this study was conducted among people with diabetes and their caregivers, the observed perspectives on diabetes speak volumes about what might be happening in the general population. This needs to be taken into consideration because the prevalence of diabetes in Tanzania is increasing [9, 12] and most people present late to a health facility due to limited knowledge and awareness of the disease [17, 21]. Similar to many other non-communicable diseases, diabetes can be delayed or prevented with the adoption of a healthier lifestyle by achieving and maintaining a healthy body weight, being physically active and eating a healthy diet [51]. However, adopting a healthy lifestyle and risk perception are believed to be important determinants of preventive behavior. There is a need for concerted efforts to increase awareness on diabetes, its risk factors, and the effectiveness of simple measures such as an appropriate lifestyle for the prevention of this disease and other NCDs.

The current study contributes to a broadened understanding of perceptions on diabetes in rural areas, particularly among people with diabetes and caregivers. The purposeful recruitment of study participants facilitated the acquisition of comprehensive information from a diverse group of people from different rural and remote villages. Although the study involved a particular group of people, people with diabetes and their caregivers, it is likely that similar perceptions on diabetes exist in other rural areas within and outside Tanzania and among different population groups, since limited knowledge and awareness on diabetes is a characteristic nature in many of the Sub Saharan African countries [48]. The study results can provide useful insights to shape strategies to raise awareness on diabetes including information on its prevention and management for better diabetes health outcomes.

## Conclusion

This qualitative study in rural Tanzania observed low awareness on diabetes causes, perceived susceptibility and preventive measures among people with diabetes and caregivers. This is concerning especially considering the severity of the disease and the preventive measures available. There is a need for policy makers, health care managers and workers to strengthen education interventions to improve patient and public knowledge on diabetes causes, and improve awareness on preventive measures such as healthy lifestyles and the adoption of healthy behaviors. This will not only contribute to reducing the social, psychological and medical burden of diabetes among people with diabetes and caregivers but also among the general population.

## Data Availability

Availability of datasets used and/or analyzed during the current study are available from the corresponding author upon reasonable request.

## Abbreviations

NCD: Non-Communicable diseases
SSA: Sub Saharan Africa
IDI: In-depth interview
